# A Nano-QSTR model to predict nano-cytotoxicity: an approach using human lung cells data

**DOI:** 10.1186/s12989-023-00530-0

**Published:** 2023-05-22

**Authors:** João Meneses, Michael González-Durruthy, Eli Fernandez-de-Gortari, Alla P. Toropova, Andrey A. Toropov, Ernesto Alfaro-Moreno

**Affiliations:** 1 NanoSafety Group, International Iberian Nanotechnology Laboratory, Braga, 4715-330 Portugal; 2 Instituto di Ricerche Farmacologiche Mario Negri IRCCS, Via Mario Negri 2, Milano, 20156 Italy

**Keywords:** Engineered nanomaterials, Computational nanotoxicology, Machine learning, Nano-QSTR, Lung nano-cytotoxicity, A549 cell line

## Abstract

**Background:**

The widespread use of new engineered nanomaterials (ENMs) in industries such as cosmetics, electronics, and diagnostic nanodevices, has been revolutionizing our society. However, emerging studies suggest that ENMs present potentially toxic effects on the human lung. In this regard, we developed a machine learning (ML) nano-quantitative-structure-toxicity relationship (QSTR) model to predict the potential human lung nano-cytotoxicity induced by exposure to ENMs based on metal oxide nanoparticles.

**Results:**

Tree-based learning algorithms (e.g., decision tree (DT), random forest (RF), and extra-trees (ET)) were able to predict ENMs’ cytotoxic risk in an efficient, robust, and interpretable way. The best-ranked ET nano-QSTR model showed excellent statistical performance with R^2^ and Q^2^-based metrics of 0.95, 0.80, and 0.79 for training, internal validation, and external validation subsets, respectively. Several nano-descriptors linked to the core-type and surface coating reactivity properties were identified as the most relevant characteristics to predict human lung nano-cytotoxicity.

**Conclusions:**

The proposed model suggests that a decrease in the ENMs diameter could significantly increase their potential ability to access lung subcellular compartments (e.g., mitochondria and nuclei), promoting strong nano-cytotoxicity and epithelial barrier dysfunction. Additionally, the presence of polyethylene glycol (PEG) as a surface coating could prevent the potential release of cytotoxic metal ions, promoting lung cytoprotection. Overall, the current work could pave the way for efficient decision-making, prediction, and mitigation of the potential occupational and environmental ENMs risks.

**Supplementary Information:**

The online version contains supplementary material available at 10.1186/s12989-023-00530-0.

## Background

Engineered nanomaterials (ENMs) based on metal oxide nanoparticles offer a wide range of promising applications, including cosmetics, electronics, sunscreens, textiles, biomedical products, and diagnostic nanodevices, among others [1–5]. Although inorganic ENMs offer multiple technological advantages and reveal exciting physicochemical properties, understanding their interaction with a biological environment is still challenging. Growing evidence demonstrates that some inorganic ENMs (e.g., CuO, ZnO, Fe_2_O_3_, CeO_2_, Ag, Au, and TiO_2_) could be potentially more toxic than their organic counterparts, such as carbon-based ENMs [2]. It is well-recognized that, from the occupational and molecular epidemiology point of view, engineered inorganic ENMs present a higher potential to induce several human lung epithelial perturbations mainly based on the intracellular increase of reactive oxygen species (ROS) [6, 7], which usually play an important role in the high prevalence of human lung nano-cytotoxicity of ENMs at the molecular, cellular, and subcellular levels (e.g., mitochondria, lysosomes, and nuclei) [7, 8].

Over the last few decades, various sampling strategies have been used to determine the ENMs occupational exposure. However, there is still no international consensus on measurement strategies, metrics, or exposure limits, as toxicity studies of ENMs have generally been conducted in non-human in vitro cell-based models. The assessment of individual human exposure to ENMs remains a critical issue despite recent innovative developments in personal measurement nanodevices [9]. In this regard, most of the research institutes that synthesize and manufacture ENMs, manage detailed action plans to mitigate the personal nano-exposure of workers, mainly by the respiratory tract [9–13]. Herein, the current nanorisk assessment paradigm was developed by the U.S. National Academy of Sciences and the Federal Government by considering four critical steps: (i) hazard identification, (ii) dose-response assessment, (iii) exposure assessment, and (iv) nano-risk characterization [14, 15]. Despite the numerous in vitro and in vivo studies to tackle the relationship between the physicochemical properties of ENMs and their nanotoxicological responses, the obtained evidence is often contradictory or nonconclusive [9].

Recently, there has been unprecedented global interest in improving human nanosafety relevance, which is possible through *in silico* models [16]. This interest has been significantly supported by increased top-down investment from central sources such as the EPA-Tox21 Consortium [17], the National Institute of Health [18], the International Organization for Standardization (ISO) [19], the European Commission through the Horizon 2020 Initiative, and the Organisation for Economic Co-operation and Development [20]. The efforts include the development of several computational models for nanotoxicology predictions [16]. Overall, *in silico* approaches are greatly beneficial to address the current concerns on ENMs nanotoxicity, as they introduce predictive animal-free technologies based on state-of-the-art machine learning (ML) methods. Newly published in Nano Today journal, Burden et al. [21] strongly suggest that by using sophisticated ML-based methodologies, which rigorously follow the 3Rs ethical principles adapted to nanosafety, it is possible to extrapolate in vitro exposure effects to explain human exposure [22, 23].

Following this idea, predictive *in silico* approaches could efficiently address Nano-Quantitative Structure-Activity/Property/Toxicity Relationships (nano-QSAR/QSPR/QSTR) of ENMs based on metal oxide nanoparticles to prevent potential human lung nano-cytotoxicity. Although the classical term to address this type of problem is QSAR, which gives a broad framework capable of integrating the most up-to-date models, in this work, the fundamental concept is nano-QSTR. Such a term derives from addressing ENMs from a toxicological point of view [24–26]. Given the complexity of the ENMs, the nano-QSTR model application is restricted by the use of physicochemical properties as nano-descriptors. Nevertheless, with the advancement of Artificial intelligence (AI) and Data Science in the last years, establishing relationships between the physicochemical properties (e.g., electronegativity, ionization potential, van der Waals radius, among others) of a complex system such as ENMs and their nanotoxicity is reasonable [27–29].

In light of the basic concept underlying the implementation of nano-QSTR predictive models, it assumes that a given set of similar structures (e.g., ENMs) have an equivalent toxicological behaviour. Thus, a subtle structural change in the ENMs composition, such as different crystallographic cores, the presence or absence of doping agents, or surface coatings, among others, should lead to a slight divergence in the toxicological paradigm. In contrast, the advances in AI and ML continue to provide large opportunities to move forward in the mechanistic understanding of nanotoxicology responses [30]. In this regard, there are several examples of transparent and understandable techniques, including multiple linear regression (MLR), partial least squares (PLS) regression, decision tree (DT), and random forest (RF), among others [31]. Besides, such algorithms should be supported by strategies that present a visual glance of the diversity and homogeneity of the data distributions and, together with the linear and non-linear correlation among the ENMs nano-descriptors, allow the exclusion of redundant information.

Therefore, this work aims to develop a novel and robust ML nano-QSTR model to predict the potential human lung nano-cytotoxicity induced by ENMs based on metal oxide nanoparticles. Moreover, the present work is an effort to pave the way for using *in silico* tools to efficiently predict ENMs potential occupational risks and make regulatory decisions in nanotoxicology and environmental health.

## Materials and methods

### Dataset

**Fig. 1:**
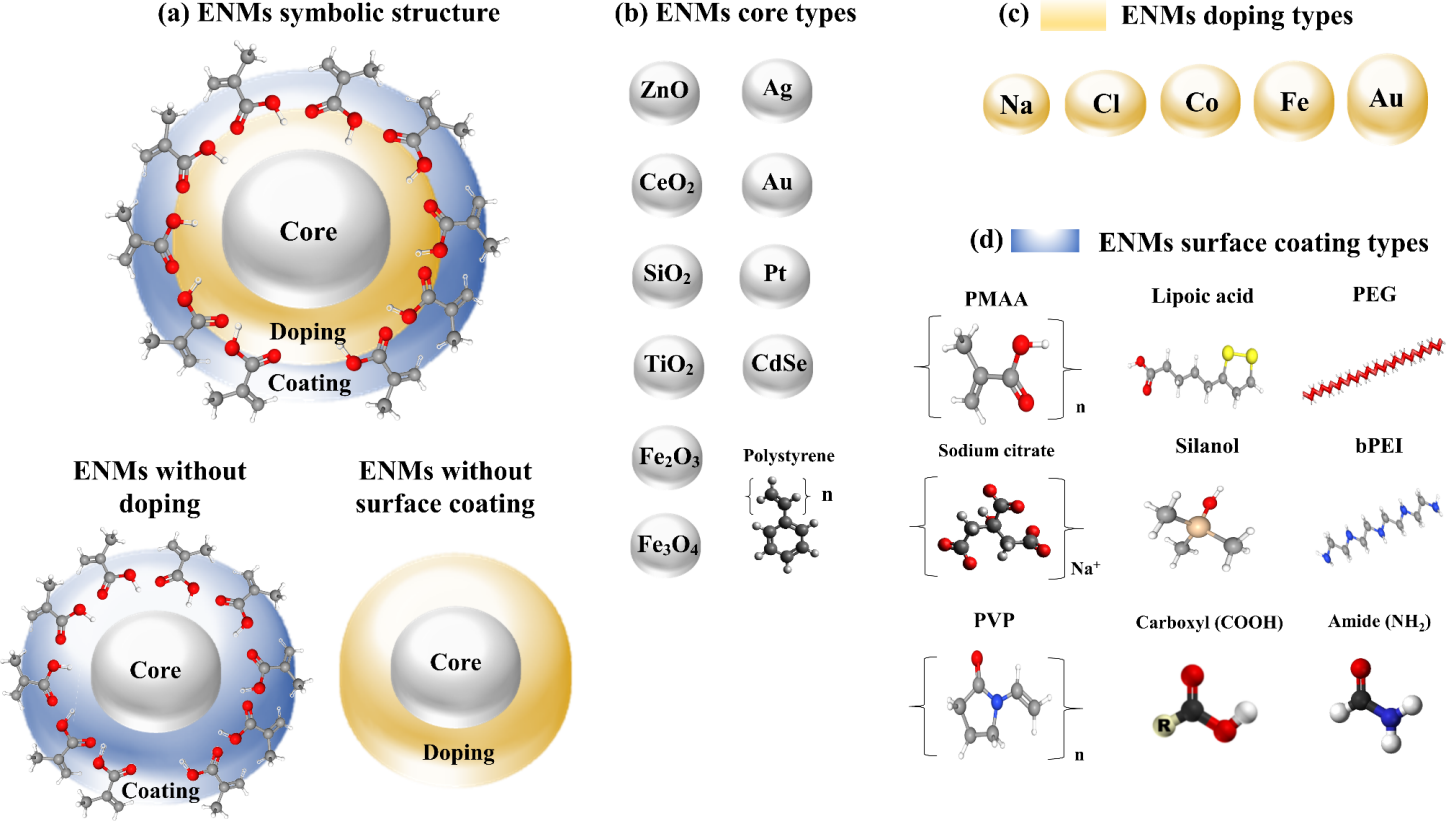
General representation of the structure and chemical composition of the engineered nanomaterials (ENMs). evaluated in this work. (**a**) Depiction of the whole composition of an ENM with the corresponding counterparts without doping and surface coating shown below; (**b**) Representation of the different ENMs core compositions; (**c**) and (**d**) Ball-and-stick model representation of the different doping and coating types evaluated for the ENMs, respectively

The data of 16 ENMs for human lung carcinoma cell line A549 have been taken from the literature [32] and recently the same data set was used by us using a quasi-SMILES approach [33]. The dataset contains 377 observations (N = 377) on cell viability (%), and covers several relevant assay conditions, such as the different composition of the core, doping, surface coating, diameter (nm), and concentrations (µg/ml) of the ENMs. An overview of the physicochemical composition of the ENMs is presented in Table 1; Fig. 1. Complete data of this subsection is available in the Supplementary Information (see Additional File 1, Table S1).

**Table 1:** A short version of the original dataset covering relevant assay conditions, such as the different physicochemical nature of the core, doping, surface coating, diameter (nm), and concentrations (µg/ml) of the ENMs along with the cell viability (%) data

ENMs ID	Core	Doping	Surface Coating	Diameter (nm)	Concentration (µg/mL)	Cell Viability (%)
1	ZnO	ND	NSC	68.90	0.01	100.00
2	ZnO	ND	NSC	68.90	1.56	104.79
3	ZnO	ND	NSC	68.90	3.13	109.64
4	ZnO	ND	NSC	68.90	6.25	112.06
5	ZnO	ND	NSC	68.90	12.50	120.16
6	ZnO	ND	NSC	68.90	25.00	47.35
7	ZnO	Na (1.5%)	NSC	5.50	0.01	100.00
8	ZnO	Na (1.5%)	NSC	5.50	6.25	94.61
9	ZnO	Na (1.5%)	NSC	5.50	12.50	93.56
10	ZnO	Na (1.5%)	NSC	5.50	25.00	93.57

ND: No doping; NSC: No surface coating

### Nano-descriptors calculation

A crucial step in developing ML nano-QSTR models is calculating the adequate nano-descriptors. Nano-descriptors are numerical forms of nanoparticle properties (e.g., electronic, physicochemical, structural, and topological) that could be used to represent ENMs and might be quantitatively associated with cytotoxicity. Moreover, nano-descriptors contain relevant information on metal, non-metal, and semimetals obtained from the periodic table and other literature sources [34]. A set of 31 nano-descriptors (e.g., Van der Waals radius of the active metal, the number of metallic elements, and the number of electrons of the active metal) was calculated using the Elemental-Descriptor (software version 1.0, Gdansk, Poland). Complete data of this subsection is available in the Supplementary Information (see Additional file 1, Table S2 and Table S3).

### Dataset pre-processing

In the context of ML nano-QSTR models, applying data pre-processing techniques represents a step that efficiently helps improve data quality by extracting relevant features from the raw data. Overall, the data pre-processing includes several procedures, such as cleaning, organizing, and structuring the data into an understandable and readable input format for building ML nano-QSTR models to predict a given biological response (i.e., cytotoxicity-induce by ENMs).

In the present study, we carried out the following pre-processing steps: (i) filter the ENMs with a diameter of less than 200 nm, reducing the number of available ENMs to 11 from the original amount of 16 ENMs, and the number of observations to N = 333 from the initial amount of N = 377; (ii) encode the categorical nano-descriptors (core, doping, and surface coating) into numerical readable inputs by using a One-Hot Encoding procedure [35]; (iii) application of a standardization procedure based on the Z-score normalization method, where the values are centered on the mean with a unit standard deviation. The standardization procedure is represented by Eq. (1), where µ is the mean of the ENMs nano-descriptor values and σ its standard deviation:

1 $${\varvec{X}}^{\varvec{{\prime }}}= \frac{\varvec{X}- \varvec{\mu }}{\varvec{\sigma }}$$

Lastly, the iv) pre-processing step was to approximate the shape of the distribution of each numerical nano-descriptor to a Gaussian distribution by applying the Yeo-Johnson transformation [36]. Complete data of this subsection is available in the Supplementary Information (see Additional file 2, Figure S1, Figure S2 and Figure S3).

### Dataset splitting

The first step was to withhold a random sample of N = 33 from a total of N = 333 to simulate an unseen dataset. Another way to think about this step is that 33 observations were unavailable to train and evaluate the ML nano-QSTR models. Afterward, the 300 observations in the dataset were randomly divided into a training subset of N = 210 (70%) and a test subset of N = 90 (30%), respectively [37]. The training subset was used to train the ML nano-QSTR models, whereas the test subset was employed to evaluate its predictive performance. Complete data of this subsection is available in the Supplementary Information (see Additional file 1, Table S4 and Table S5).

### ML nano-QSTR model development

Considering the trade-off between ML nano-QSTR model performance and its interpretability, the ENMs nano-descriptors were combined by simple arithmetical operations and converted into new statistically significant nano-descriptors. For instance, two ENMs nano-descriptors were multiplied to be more preponderant in explaining the data variance than the same two ENMs nano-descriptors separately. The next step was to reduce the dimensionality of the data, reduce the computational cost, and minimize the redundancy between the ENMs nano-descriptors. Three key operations were performed, namely (i) remove the ENMs nano-descriptors with a low variance; (ii) remove highly inter-correlated ENMs nano-descriptors; (iii) implement a combination of various permutation importance techniques to achieve the final subset of ENMs nano-descriptors [38], such as Shapley additive explanations (SHAP), which explains the contribution of each ENMs nano-descriptor to the ML nano-QSTR model [39]. Afterward, several tree-based algorithms were used to make the ML pipeline more transparent and interpretable, such as decision tree (DT), random forest (RF), and extra-trees regressor (ET) [31]. Complete data of this subsection is available in the Supplementary Information (see Additional file 1, Table S6).

### ML nano-QSTR model validation

In the present study, we followed the principles of the Organization for Economic Co-operation and Development (OECD) concerning model validation, which establishes that a reliable model should present appropriate goodness-of-fit measures, robustness, and predictivity performance [40]. In this regard, there are two methods to evaluate the goodness of a model: (i) internal and (ii) external validation. The (i) internal validation evaluates the fitting of the model on existing data (training set); the (ii) external validation evaluates future data, i.e., how reliable the model can predict new data (test set and unseen set). Here, the (i) internal validation of the regression-based models was determined based on several statistical metrics such as determination coefficient (R^2^), determination coefficient based-metrics (Q^2^_LOO_), root-mean-square error (RMSE), mean absolute error (MAE), and coefficient of concordance (CCC). Moreover, the robustness of the model was represented by a 5-fold cross-validation process [41]. The (ii) external validation of the models was determined using similar statistical parameters, such as R^2^_ext_, Q^2^_F1_, Q^2^_F2_, RMSE, MSE, and CCC. All statistical metrics were computed via DTC Lab Xternal Validation Plus (software version 1.2, India) [42].

### Applicability domain

According to the OECD third principle [40], a QSAR model to predict a given biological response (i.e., ENMs cytotoxicity) should be associated with a defined applicability domain (AD). The AD is a theoretical region of the chemical space that contains both model nano-descriptors and modelled responses, in which the model makes predictions with a given reliability [43]. Herein, the AD was calculated using a standardization approach and retrieved via DTC Lab Applicability Domain Calculator (software version 1.0, India). For calculating the AD, Eq. (2) was used:

2 $${S_{new}}_{\left( k \right)} = \overline{\overline {{S_k}}} + 1.28 \times {\sigma _{{S_k}}}$$

Where S_new (k)_ is S_new_ value for ENM_k_, S̅_k_ is the mean of the standardized nano-descriptors for ENM_k_ (from the training, test, or unseen set), and σ_Sk_ is the standard deviation of standardized nano-descriptors for ENM_k_ (from the training, test, or unseen set). Overall if the S_new (k)_ is lower or equal to 3, then the ENM_k_ is not an outlier (if in the training set) or is within the AD (if in the test or unseen set) [43].

### Summary

**Fig. 2:**
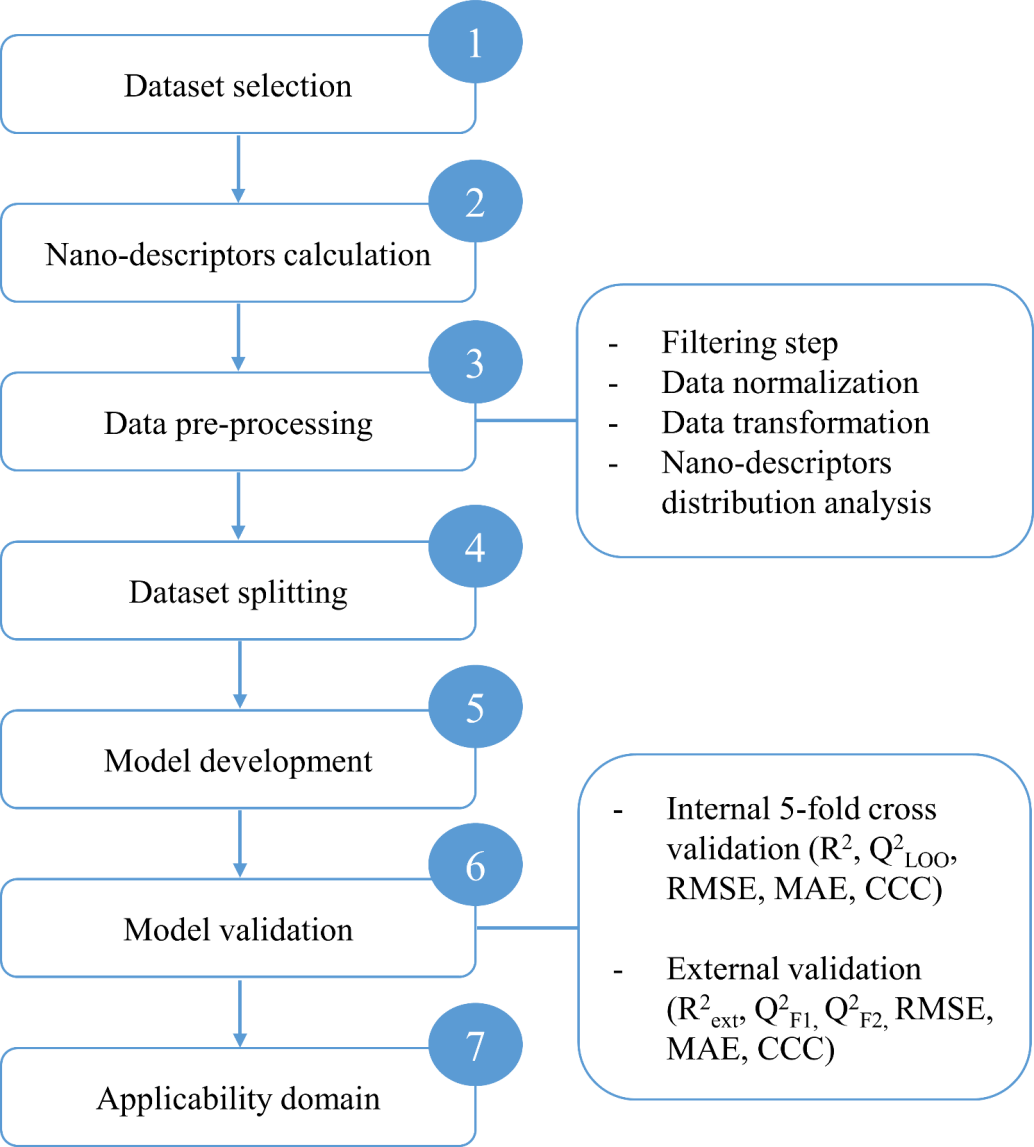
Overview of ML nano-QSTR model approach to predict the potential human lung nano-cytotoxicity induced by ENMs based on metal oxide nanoparticles

An overview of the implemented data-driven approach is presented in Fig. 2. All algorithms were implemented in Python (software version 3.9), using libraries such as Pycaret (software version 2.3.8) and scikit learn (software version 0.23.2). Altogether, the experiments allowed the extraction of valuable insights from the dataset and provided the baseline to construct the ML nano-QSTR models. The definition of AD made it possible to understand the limitations and boundaries where the predicted values can be trusted with confidence.

## Results

### Nano-descriptors distribution and diversity

Based on the data of 16 engineered nanomaterials (ENMs), under different experimental conditions, a ML nano-QSTR model with cell viability endpoint as the dependent variable was established. As previously mentioned, the ultimate goal of the developed model was to predict the potential human lung nano-cytotoxicity.

**Fig. 3:**
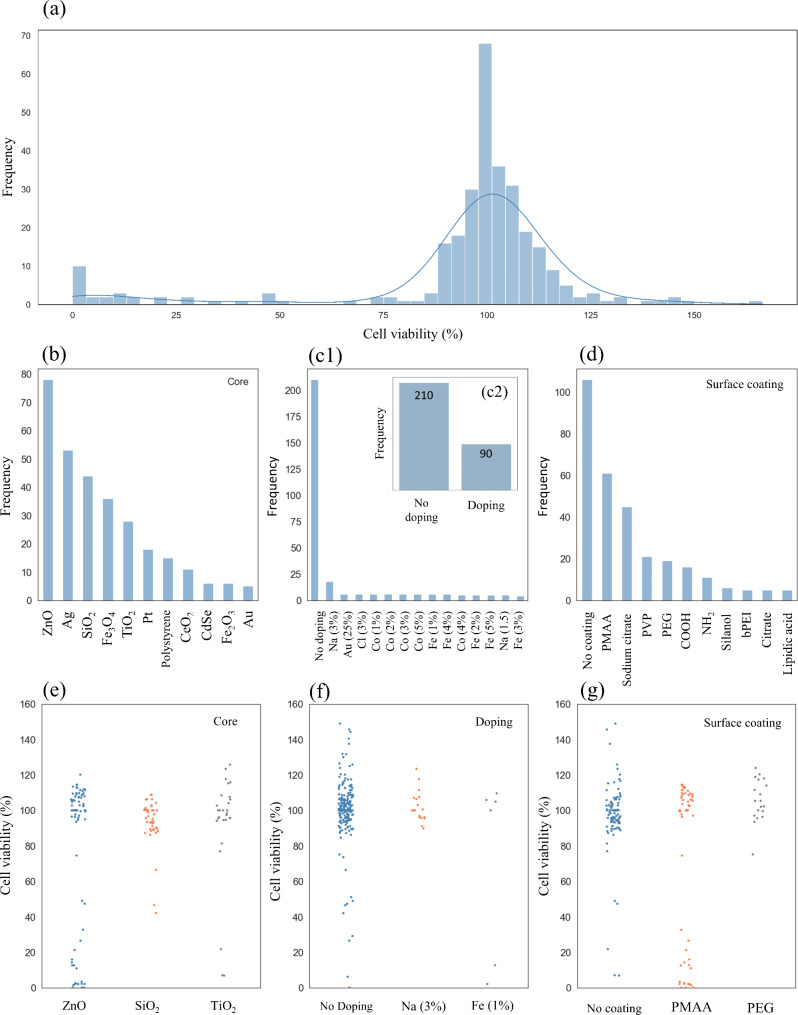
Dataset overview. (**a**) Cell viability (%) frequency distribution; (**b**), (**c1**), (**c2**), and (**d**) Diversity of core, doping, and surface coating nanomaterials composition; (**e**), (**f**), and (**g**) Cell viability (%) variation trend with representative examples of each nano-descriptor

As the application of any data-driven algorithm requires a comprehensive understanding of the data, one of the first concerns was to consider the differences in the structure and chemical composition of the ENMs, extract valuable insights from the dataset and provide a baseline to construct the ML nano-QSTR model. In this regard, Fig. 3 presents an overview of the dataset characteristics, such as the endpoint frequency distribution, and the diversity of core, doping, and surface coating nanomaterials composition.

It is not uncommon to find a skewed frequency distribution for ENMs nano-descriptors from nanotoxicological experimental data, i.e., some types of structural attributes appear much more frequently [44]. In this regard, the evaluated endpoint (cell viability) follows a normal distribution, predominantly ranging from 75 to 125%, with a mean value of 96% and a standard deviation of 23% (Fig. 3(a)). Regarding the composition of the ENMs, based on their frequency distribution, the core and the surface coating features present a qualitative and quantitative diversity, with the most prevalent core types being Zn, Ag, SiO_2_, and surface coating compositions being the ENMs without surface coating, and the ENMs coated with PMAA, and sodium citrate (Fig. 3(b) and Fig. 3(d)). The frequency distribution of the different doping types is predominantly represented by the absence of doping in the ENMs and may seem skew toward that direction (Fig. 3(c1)). However, it is important to note that for ENMs with and without doping, the frequency distribution of both types is much more balanced (Fig. 3(c2)). To complement this analysis, Fig. 3(e) to Fig. 3(g) present the trend of the cell viability variation with representative examples of each nano-descriptor. Despite the existence of specific ENMs compositions that significantly vary the cell viability, the tendency of variation agrees with the mean and standard deviation previously mentioned. A similar frequency distribution analysis was performed on the set of 31 ENMs nano-descriptors, such as the number of metallic elements, the Van der Waals radius of the active metal, and the number of electrons of the active metal, among other examples (see Additional file 2, Figure S4). Overall, each nano-descriptor depicts a high degree of diversity by presenting several attributes that can span the ENMs structural space.

### ML nano-QSTR model performance and validation

Through the dataset exploration and characterization conducted in the previous section, the richness of the dataset was assured, providing the basis for the development of the ML nano-QSTR model. In this particular problem, three interpretable learning algorithms, including decision tree (DT), random forest (RF), and extra-trees regressor (ET) are presented. Although DT, RF, and ET belong to the same family of learning algorithms, i.e., tree-based models that use conditional statements to make predictions, DT is the simplest. Therefore, it is expected that DT presents slightly lower statistical metrics than RF and ET. The use of such an algorithmic family is in agreement with recent studies that point out that an interpretable learning algorithm is more valuable to experimentalists than a highly predictive but black-box model since its interpretation is complex and non-trivial [31, 45]. To verify the reliability and robustness of the developed models, Table 2 presents an overview of the statistical metrics for training, validation, and test sets.

**Table 2:** ML nano-QSTR models performance for training, validation, and test sets

Model	Subset	R^2^	R^2^_ext_	Q^2^_LOO_	Q^2^_F1_	Q^2^_F2_	RMSE	MAE	CCC
DT	Training	0.953	-	-	-	-	6.147	3.840	0.976
	Validation	0.733	-	0.730	-	-	14.103	8.441	0.851
	Test	-	0.765	-	0.765	0.765	13.264	7.139	0.868
RF	Training	0.965	-	-	-	-	5.299	2.963	0.981
	Validation	0.768	-	0.767	-	-	13.266	7.908	0.866
	Test	-	0.790	-	0.789	0.789	12.573	6.879	0.881
ET	Training	0.953	-	-	-	-	6.158	3.912	0.975
	Validation	0.798	-	0.797	-	-	12.054	7.294	0.889
	Test	-	0.788	-	0.788	0.788	12.580	6.603	0.883

DT: decision tree; RF: random forest; ET: extra-trees regressor; R^2^: determination coefficient (R^2^_ext_ for external validation); Q: determination coefficient based-metrics (Q^2^_LOO_ for internal validation; Q^2^_F1_ and Q^2^_F2_ for external validation); RMSE: root-mean-square error; MAE: mean absolute error; CCC: coefficient of concordance.

Regarding the evaluation of the developed models on existing data, i.e., training subset, DT, RF, and ET present an R^2^ between 0.95 and 0.96, highlighting their high statistical performance in learning the behavior of the training ENMs. Concerning DT, RF, and ET internal validation, the 5-fold cross-validation process enhanced their robustness as R^2^ for each model is between 0.7 and 0.8. As previously mentioned, with the slight increase in model complexity, there is an increment in the statistical performance as DT presents an R^2^ of 0.73, RF of 0.76, and ET of 0.79. Overall, the internal validation of each model is guaranteed by R^2^ and Q^2^_LOO_ to be in the same order of magnitude. As for the evaluation of the developed models on new data, i.e., test subset, DT presents an R^2^_ext_ of 0.76, while RF and ET present an R^2^_ext_ of 0.79. This statistical parameter is complemented by the determination coefficient based-metrics (Q^2^_F1_ and Q^2^_F2_), which depict similar values. Altogether, both internal and external validation confirms that the developed models have the potential to reliably predict A549 cell line viability.

**Fig. 4:**
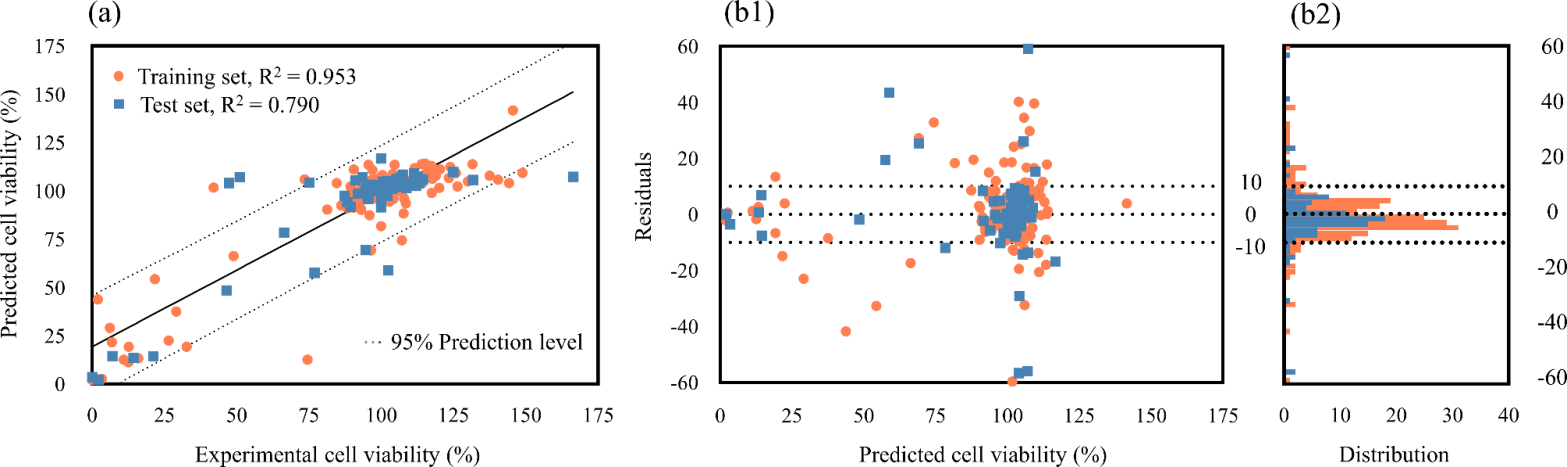
Overview of experimental and predicted cell viability (%) by ET nano-QSTR model for training and test sets. (**a**) Scatter plot representing the experimental cell viability (%) as a function of the predicted cell viability (%). The straight line illustrates the perfect agreement between experimental and calculated values. The dashed lines represent the 95% prediction level. (**b1**) Scatter plot representing the predicted cell viability (%) as a function of the residuals; (**b2**) Frequency distribution of the predicted cell viability (%). The dashed lines represent the residuals that are between − 10 and 10

From a general point of view, the key performance indicator in selecting the model to be used in the final stage of prediction was the internal validation subset. Such a decision relies on ET presenting R^2^ and Q^2^-based metrics higher than DT in 8% and RF in 4%, while the training and test set statistical parameters are in the same order of magnitude. To corroborate this analysis, Additional file 2, Figure S5 presents the learning curves for the ET nano-QSTR model for training and validation sets, highlighting a slight trade-off between bias and variance, which could be adjusted by having more training instances. Thus, Fig. 4 presents an overview of the agreement between experimental and predicted cell viability values by the ET model for training and test sets.

Through the exploration of Fig. 4(a), it is possible to quantitative and qualitatively highlight the strong correlation between the observed and predicted cell viability values, as most ENMs are within the 95% prediction level range. Such analysis is complemented by Fig. 4(b), which enhances that training and test data points approximately follow a symmetrical distribution, tending to cluster towards the middle of the plot, around lower values of the y-axis (e.g., most of the residuals are between − 10 and 10).

### Nano-descriptors interpretation

**Fig. 5:**
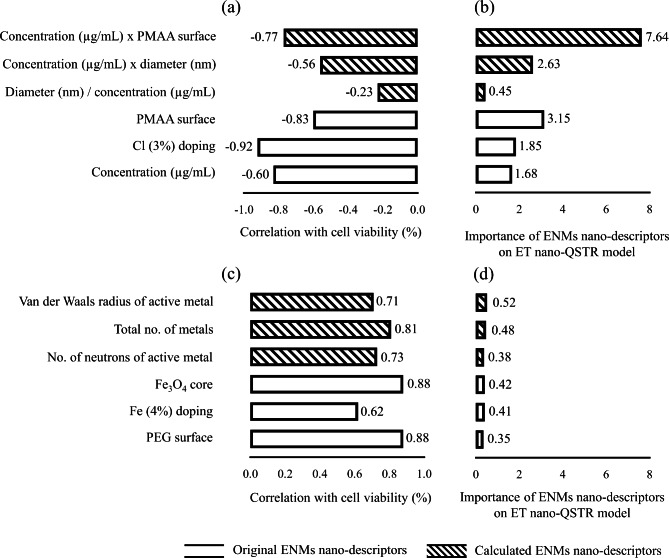
Summary of representative nano-descriptors. (**a**) and (**c**) Negatively and positively correlated nano-descriptors with cell viability (%); (**b**) and (**d**) Nano-descriptors effect on ET nano-QSTR model performance

Bearing in mind that the final aim of the present work is to predict the potential nano-cytotoxicity induced by ENMs on human lung carcinoma cells, it is fundamental to understand the correlation between the nano-descriptors and the cell viability. Moreover, it is noteworthy to highlight the most significant nano-descriptors in the ET nano-QSTR model performance. Thus, Fig. 5 presents an overview of some representative nano-descriptors and their correlation with cell viability.

Through the exploration of Fig. 5(a), it is possible to identify two distinct groups of nano-descriptors that present a negative correlation with cell viability. One group depicts nano-descriptors retrieved from the original dataset, such as PMAA surface coating, Cl (3%) doping, and ENMs concentration. The other group describes a set of nano-descriptors that were calculated to become more preponderant in explaining variances in the cell viability data, such as the mathematical combination of ENMs concentration and PMAA surface, ENMs concentration, and ENMs diameter. Overall, Fig. 5(a) highlights nano-descriptors that tend to decrease cell viability and might promote cytotoxicity at the cellular level. As for nano-descriptors that present a positive correlation with the endpoint, Fig. 5(c) highlights Fe_3_O_4_ core, Fe (4%) doping, and polyethylene glycol (PEG) surface coating as the group of nano-descriptors retrieved from the original dataset. In a similar analysis, the other group describes a set of nano-descriptors obtained from the Elemental descriptor calculator, such as the Van der Waals radius of the active metal, the number of metallic elements, and the number of electrons of the active metal. Comprehensively, Fig. 5(c) enhances nano-descriptors that tend to increase cell viability and might avoid cytotoxicity at the cellular level.

From a modelling point of view, Fig. 5(b) and Fig. 5(d) represent a fundamental aspect as both figures highlight the set of nano-descriptors that contributed the most to the model performance. Interestingly, the nano-descriptors that present a negative correlation with the endpoint are the most relevant from the entire set. An in-depth analysis of Fig. 5(b) shows that the nano-descriptor with the highest contribution on the model performance results from the mathematical combination of the ENMs concentration and PMAA surface coating.

Considering that ENMs surface area is described as a central factor related to the toxic potential of particulate matter, we did explore if this descriptor could play a relevant role in our model. As detailed above, several nano-descriptors linked to the surface coating reactivity properties and diameter of the ENMs were identified as some of the most relevant characteristics to predict human lung nano-cytotoxicity. To mathematically calculate the surface area of the ENMs, it is necessary to perform some assumptions, namely (i) the ENM is a perfect sphere, and (ii) the ENM size is the diameter of the sphere. Therefore, even though indirectly, the model does consider the surface area of the ENMs as a driving factor for nano-cytotoxicity. All the assumptions and mathematical equations to calculate the surface area are explained in detail by Shin et al. [32]. Complete data on the direct influence of ENMs surface area on model performance is available in the Supplementary Information (see Additional file 1, Table S7, and Additional file 2, Table S1).

Taking advantage of such knowledge, the ET nano-QSTR model was retrained with the ENMs nano-descriptors identified in Fig. 5 and applied to new data, which was not used to develop and validate the model. Table 3 presents an overview of the statistical metrics of ET nano-QSTR model when applied to the unseen subset.

**Table 3:** ET Nano-QSTR model performance for unseen subset

Model	Subset	R^2^_ext_	Q^2^_F1_	Q^2^_F2_	RMSE	MAE	CCC
ET	Unseen	0.927	0.929	0.925	4.375	3.528	0.961

ET: extra-trees regressor; R^2^_ext_: determination coefficient for external validation; Q^2^_F1_ and Q^2^_F2_: determination coefficient based-metrics for external validation; RMSE: root-mean-square error; MAE: mean absolute error; CCC: coefficient of concordance.

Table 3 shows a significant increase in ET nano-QSTR model performance as the R^2^_ext_ increased from 0.79 to 0.93, representing an increase of 18%. Another interesting observation is the similar order of magnitude between the R^2^_ext_ and Q^2^-based metrics. Additionally, the RMSE, MAE, and CCC performance metrics increased from 12.58 to 4.37, 6.60 to 3.52, and 0.883 to 0.96, representing an increase of 65%, 47%, and 8%, respectively. Overall the developed model presents a strong, reliable, and robust performance in predicting cell viability.

### Applicability domain of the proposed model

However, it is paramount to identify the border of the optimum prediction space, i.e., applicability domain (AD). From a general point of view, the developed ET nano-QSTR model is valid in a chemical space where the ENMs possess structural and physicochemical properties similar to the ENMs used to train and validate the model. Otherwise, the ENMs might be considered outliers or even out of the AD. It is significant to mention that all the studied ENMs are within the AD. Detailed values of the AD of the ENMs are depicted in the Supplementary Information (see Additional file 1, Table S8).

## Discussion

Although human lung nano-cytotoxicity induced by ENMs based on metal oxide nanoparticles is among the current occupational and environmental concerns, it remains unexplored and under-researched. The current standard in vitro and in vivo models used to evaluate such a type of nano-cytotoxicity are time-consuming, costly, and could involve many ethical concerns in animal experimentation. In this sense, this work presents an ET nano-QSTR model to assist experimental scientists by providing a mechanistic interpretation learned from data of 16 ENMs for the A549 cell line [40, 46].

The mechanistic interpretation regards the set of optimal nano-descriptors, i.e., the most significant nano-descriptors in the model performance, and considers if the nano-descriptors are (i) negatively or (ii) positively correlated with the cell viability [47, 48]. Then, three well-recognized nanotoxicological mechanisms are used to complement such interpretation, including (i) the ENMs core type- and diameter-dependent release of cytotoxic metal ions (e.g., Fe^2+^, Fe^3+^, Ag^+^, Au^+^, Ti^2+^, Cd^2+^) from the ENMs core reactive surface, which could promote redox-homeostasis perturbations, (ii) the physio-pathological increase of intracellular reactive oxygen species (ROS) levels by mitochondrial dysfunction, and (iii) the nano-bio interaction with binding sites of key molecular targets, such as the human lung epithelial proteins and multiprotein junctional complexes that form the selective permeability barrier of the human lung epithelial, which may induce barrier dysfunction [7, 32, 49, 50].

An in-depth analysis of Fig. 5, which identifies six negatively and positively correlated nano-descriptors with cell viability, suggests that the presence of PEG as a surface coating of a Fe_3_O_4_ core significantly enhances cell viability and inversely attenuates human lung nano-cytotoxicity. As PEG presents excellent pharmacokinetic properties based on absorption, distribution, metabolism, and excretion (ADME), its presence as a surface coating could significantly reduce the potential release of cytotoxic ions (Fe^2+^) from the Fe_3_O_4_ core. Overall, such behavior is congruent with the literature, as the surface-dependent release of cytotoxic metal ions has been well documented in previous experimental works [51–54].

In this regard, the generated divalent Fe^2+^ ions from the ENMs based on metal oxide nanoparticles tend to increase the intracellular concentration of oxygen-free-radical groups (e.g., hydroxyl radical). Such mechanism is explained due to the occurrence of the Fenton-Haber-Weiss reaction at the subcellular level, which is directly associated with molecular lung cytotoxic mechanisms (e.g., mitochondria dysfunction promoted by Fe^2+^ ions) [55–59]. Therefore, the presence of PEG as a surface coating significantly contributes to the inhibition of these cytotoxic signaling pathways.

In opposition to the previously described behavior of PEG, the presence of PMAA as a surface coating or the Cl^−^ (3%) as a doping condition tend to decrease cell viability, as they intensify the potential release of cytotoxic metal ions (e.g., Fe^2+^, Fe^3+^, Ag^+^, among others) from the inorganic ENMs core or doping composition [51–54, 60]. Such evidence is corroborated by the ET nano-QSTR model, as the most influential nano-descriptor to the model performance derives from the arithmetic combination of the ENMs concentration and PMMA surface coating. Furthermore, the nano-descriptor also presents a high negative correlation (R^2^ = − 0.83) with cell viability and may be inversely linked with human lung nano-cytotoxicity.

Besides, all the core-based ENMs nano-descriptors, such as the number of metallic elements (R^2^ = 0.81), the Van der Waals radius of the active metal (R^2^ = 0.71), and the number of electrons of the active metal (R^2^ = 0.73), are positively correlated with the cell viability. Such structural attributes are directly associated with the metal core-based reactive properties when the ENMs are found in their pristine form and have an intrinsic cytotoxic potential according to the diameter and charge of the inorganic core. Nonetheless, such nano-descriptors do not contribute to intensifying the cytotoxicity from the *in silico* point of view. A reasonable explanation behind this behavior focuses on the presence of a PEG surface coating that stabilizes the ENMs’ chemical surface reactivity and avoids the occurrence of direct nano-bio interactions of the metal oxide core with binding sites of the target proteins forming the human lung epithelial (i.e., A549 cells target proteins).

It is well-established that a decrease in the diameter of the metal oxide nanoparticles contributes to a significant increase in their ability to access lung cell compartments, including mitochondria, lysosomes, and nuclei. Therefore, the concentration of metal oxide nanoparticles in such organelles will increase and play a fundamental role in lung epithelial barrier dysfunction [7]. More importantly, the diameter decrease of the evaluated metal oxide nanoparticles could contribute to an exponential increase in the number of reactive atoms expressed on the face-based crystallographic planes of the ENMs core. Such a decrease simultaneously promotes the core chemical reactivity and its potential capacity to interact with relevant lung tissues and cells [7, 61, 62]. The proposed ML nano-QSTR model corroborates this information through the negatively correlated nano-descriptors that result from the mathematical combination of ENMs concentration and ENMs diameter.

Overall, the current mechanistic interpretation brings a novel contribution to assist experimental scientists in understanding and analyzing nanotoxicological data. Nonetheless, achieving a broader domain of applicability and trustability in nano-QSTR models is still a challenge. Therefore, it is necessary to start or continue to collect human lung experimental data standardly, i.e., to implement the findability, accessibility, interoperability, and reusability (FAIR) data principles [63]. In this regard, it is crucial to highlight that data concerning (i) the potential release of cytotoxic metal ions through time and consequently, (ii) the actual concentration of ENMs that reach the cells were not included in the modeling procedure due to the unavailability of such data for human lung cells.

Indeed, using advanced computational models to predict the effective cellular dose is fundamental to understanding the interaction of submerged materials with biological systems. Recently, DeLoid et al. [64] explored both three-dimensional computational fluid dynamics (CFD) and a newly-developed one-dimensional Distorted Grid (DG) model to predict the delivered dose metrics for submerged ENMs in culture media. The last model was later used and validated in a study by Kowoll et al. [65] to predict the deposition of particles on cellular and intercellular human lung surfaces. Interestingly, the authors highlight both model capabilities and limitations, specifically regarding the spatial distribution of particles on heterogeneous surfaces, which is the case in our study. Such considerations are even more relevant since Kowoll et al. performed the experiments in a human lung cell line (i.e., A549 cells) that fit with the same biological model considered in our *in silico* study.

Therefore, in future works, we plan to address these limitations by incorporating computational particokinetics models to estimate the relationship between the release of cytotoxic metal ions through time, the relevant in vitro dose criteria for the dosimetry of ENMs, and their influence on the shape of the dose-response curve [66]. Even with all the challenges, a prospective nano-QSTR model should be performed in a useful way that could lead and orient experimental scientists to decision-making processes about nanotoxicological data.

## Conclusions

An ML nano-QSTR model was successfully developed to predict the potential human lung nano-cytotoxicity induced by ENMs based on metal oxide nanoparticles. Results demonstrated that using tree-based learning algorithms (e.g., extra-trees regressor) allowed the development of a simple, interpretable, and robust nano-QSTR model, as ET presented R^2^ and Q^2^-based metrics of 0.95, 0.80, and 0.79 for training, internal validation, and external validation subsets. By taking advantage of the advances in AI and ML, which continue to provide opportunities to move forward in the mechanistic understanding of nanotoxicology responses, we could identify the six most significant nano-descriptors in the model performance. Therefore, we could understand if the nano-descriptors are (i) negatively or (ii) positively correlated with the cell viability. As for the (i) negatively correlated, a decrease in the diameter of the metal oxide nanoparticles contributes to a significant increase in their ability to access lung cell compartments, thus promoting lung epithelial barrier dysfunction. As for the (ii) positively correlated, the presence of PEG as a surface coating significantly stabilizes the ENMs’ chemical surface reactivity and avoids the potential release of cytotoxic ions, promoting cell viability and inversely attenuating human lung nano-cytotoxicity. By exploiting such knowledge, the ML nano-QSTR model was retrained with the most significant nano-descriptors and applied to new data (e.g., unseen subset), allowing the increase of R^2^ and Q^2^-based metrics from 0.79 to 0.92. Based on these findings, the present work may pave the way to possibly predict ENMs’ potential occupational risks and make regulatory decisions in nanotoxicology and environmental health.

## Electronic supplementary material

Below is the link to the electronic supplementary material

**Additional file 1. Table S1** Original dataset. **Table S2** List and description of calculated nano-descriptors. **Table S3** Dataset with calculated nano-descriptors. **Table S4** Dataset for modelling after the filtering step. **Table S5** Unseen subset after the filtering step. **Table S6** Training and test data after nano-descriptors selection. **Table S7** Dataset assuming data normalization by ENMs surface area. **Table S8** Applicability domain of the proposed ML nano-QSTR model.

**Additional file 2. Figure S1** ENMs diameter (nm) frequency distribution (a) before and (b) after the filtering step. **Figure S2** Cell viability (%) frequency distribution (a) before and (b) after the filtering step. **Figure S3** General representation of data normalization and transformation pre-processing steps for ENMs diameter and concentration. **Figure S4** Dataset overview. (a), (b), and (c) Diversity of a set of nano-descriptors obtained from the Elemental descriptor calculator; (e), (f), and (g) Cell Viability (%) variation trend with representative examples of each nano-descriptor. **Figure S5** Learning curves for ET nano-QSTR model for training and validation sets according to (a) determination coefficient (R2) and (b) root-mean-square error (RMSE). **Table S1** ET nano-QSTR model performance for training, validation, test, and unseen sets assuming data normalization by ENMs surface area.

## Data Availability

The datasets supporting the conclusions of this article are included in the Supplementary Information. The python script can be retrieved from the corresponding author upon reasonable request.

## List of abbreviations

AI: Artificial Intelligence
AD: Applicability Domain
ADME: Absorption, Distribution, Metabolism, and Excretion
CCC: Coefficient of Concordance
DT: Decision Tree
ET: Extra-trees
ENMs: Engineered Nanomaterials
FAIR: Findability, Accessibility, Interoperability, and Reusability
ISO: International Organization for Standardization
ML: Machine Learning
MLR: Multiple Linear Regression
OECD: Organization for Economic Co-operation and Development
PEG: Polyethylene Glycol
PLS: Partial Least Squares
Q^2^: Determination Coefficient Based-Metrics
QSAR: Quantitative-Structure-Activity Relationship
QSPR: Quantitative-Structure-Property Relationship
QSTR: Quantitative-Structure-Toxicity Relationship
RF: Random Forest
R^2^: Determination Coefficient
ROS: Reactive Oxygen Species
RMSE: Root-Mean-Square Error
SHAP: Shapley Additive Explanations
