# Supplementary material for: A Nano-QSTR model to predict nano-cytotoxicity: an approach using human lung cells data
Source: Part Fibre Toxicol. 2023 May 22;20:21. doi: 10.1186/s12989-023-00530-0 (PMC10201760; doi:10.1186/s12989-023-00530-0)
Supplement: Supplementary file 2 — Additional file 2. Figure S1 ENMs diameter (nm) frequency distribution (a) before and (b) after the filtering step. Figure S2 Cell viability (%) frequency distribution (a) before and (b) after the filtering step. Figure S3 General representation of data normalization and transformation pre-processing steps for ENMs diameter and concentration. Figure S4 Dataset overview. (a), (b), and (c) Diversity of a set of nano-descriptors obtained from the Elemental descriptor calculator; (e), (f), and (g) Cell Viability (%) variation trend with representative examples of each nano-descriptor. Figure S5 Learning curves for ET nano-QSTR model for training and validation sets according to (a) determination coefficient (R2) and (b) root-mean-square error (RMSE). Table S1 ET nano-QSTR model performance for training, validation, test, and unseen sets assuming data normalization by ENMs surface area. [file 12989_2023_530_MOESM2_ESM.docx]

**RESEARCH**

**A Nano-QSTR model to predict nano-cytotoxicity: an approach using human lung cells data**

João Meneses^1^, Michael González-Durruthy^1^, Eli Fernandez-de Gortari^1^, Alla P. Toropova^2^, Andrey A. Toropov^2^, Ernesto Alfaro-Moreno^1^(*)

^1^ NanoSafety Group, International Iberian Nanotechnology Laboratory, 4715-330 Braga, Portugal

^2^ Instituto di Ricerche Farmacologiche Mario Negri IRCCS, Via Mario Negri 2, 20156 Milano, Italy

* Correspondence: ernesto.alfaro@inl.int

**Supplementary Information**

**Additional File 2**


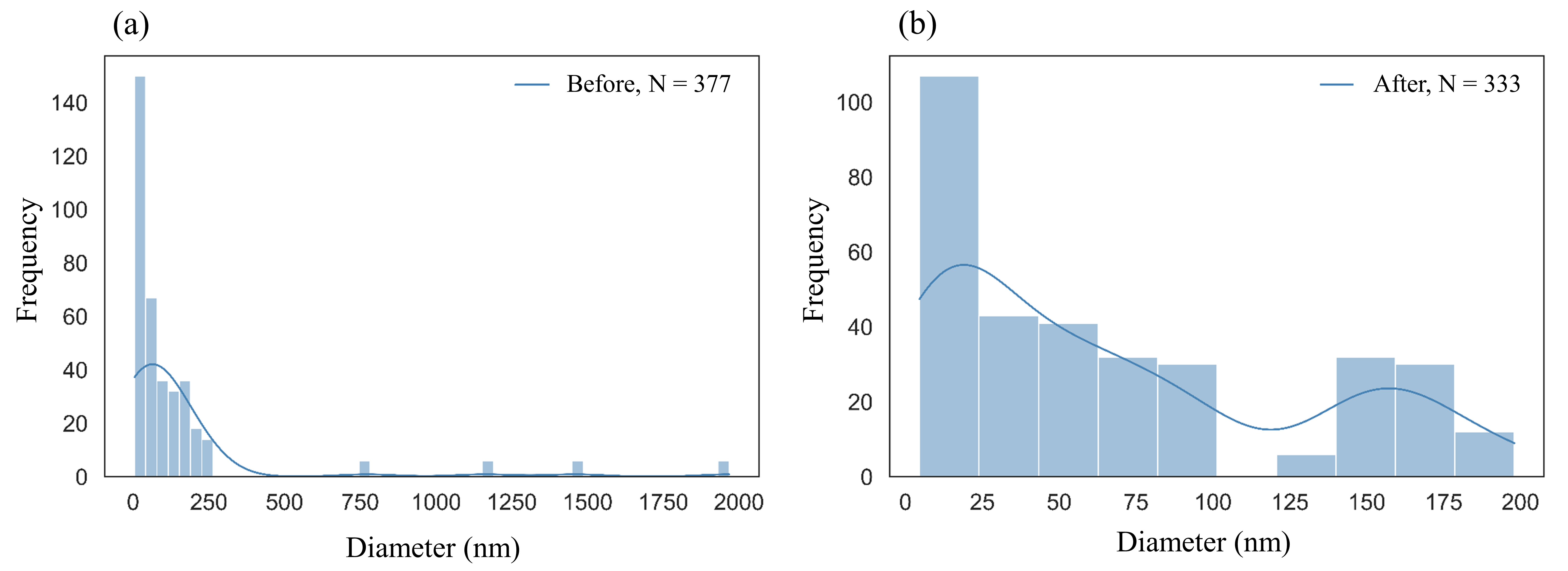


**Figure S1** ENMs diameter (nm) frequency distribution (a) before and (b) after the filtering step.


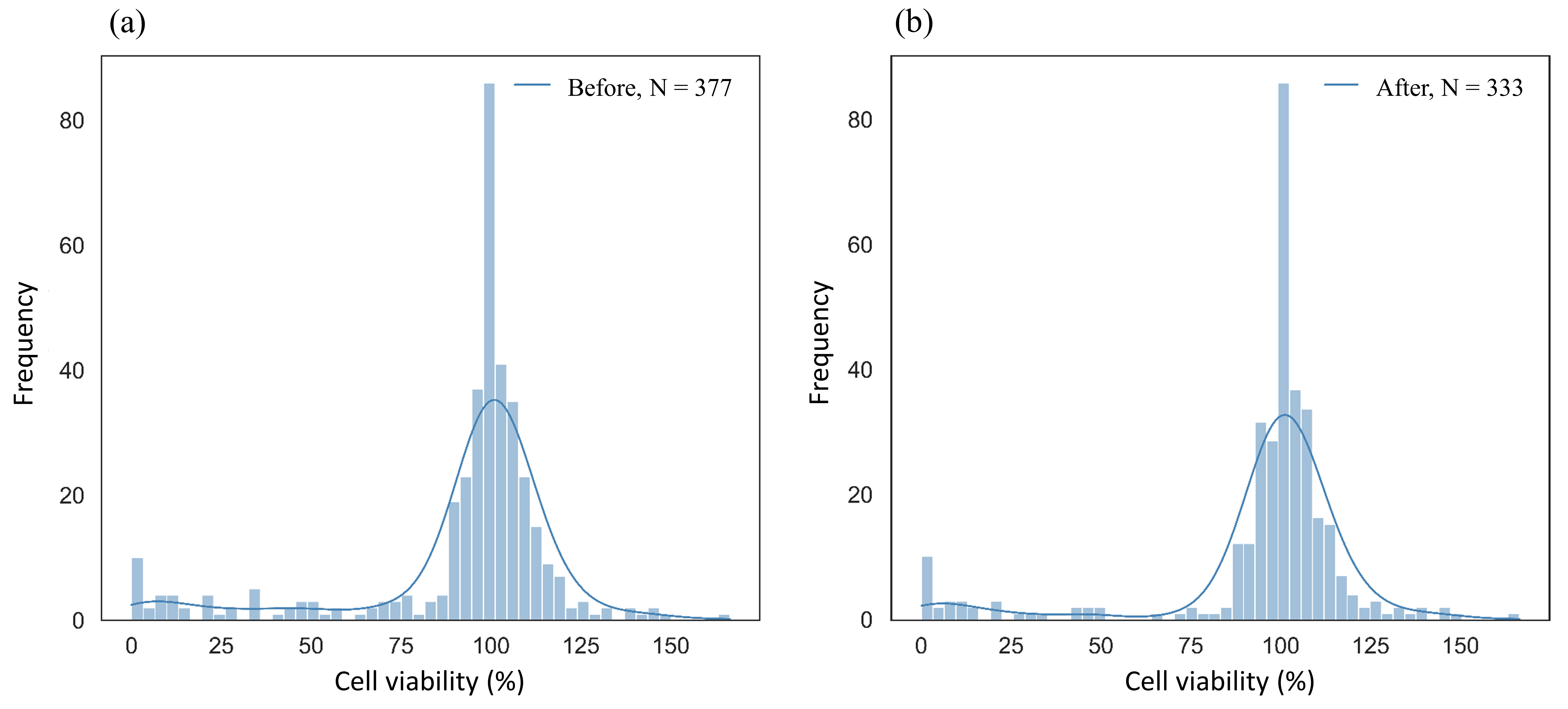


**Figure S2** Cell viability (%) frequency distribution (a) before and (b) after the filtering step.


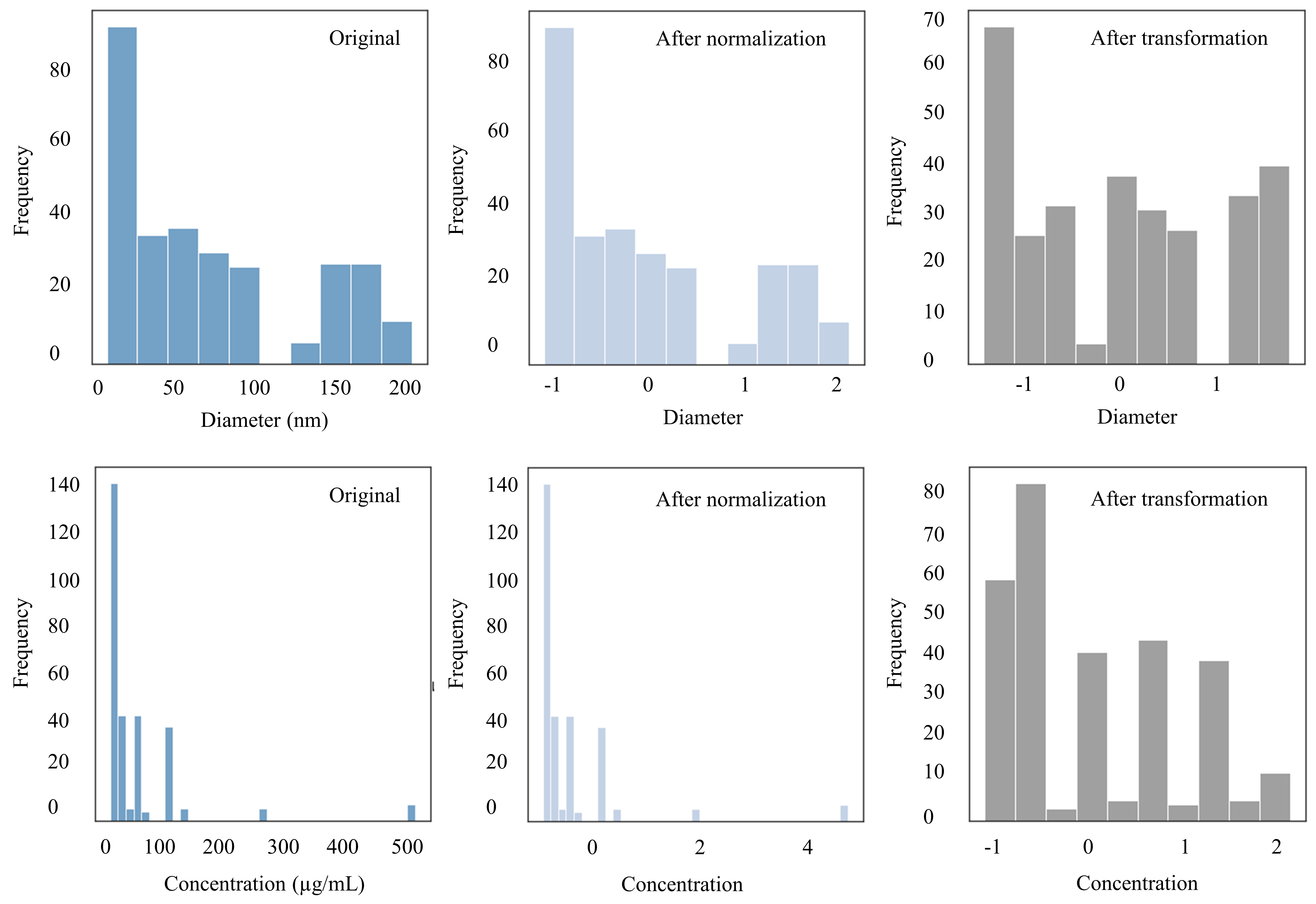


**Figure S3** General representation of data normalization and transformation pre-processing steps for ENMs diameter and concentration.


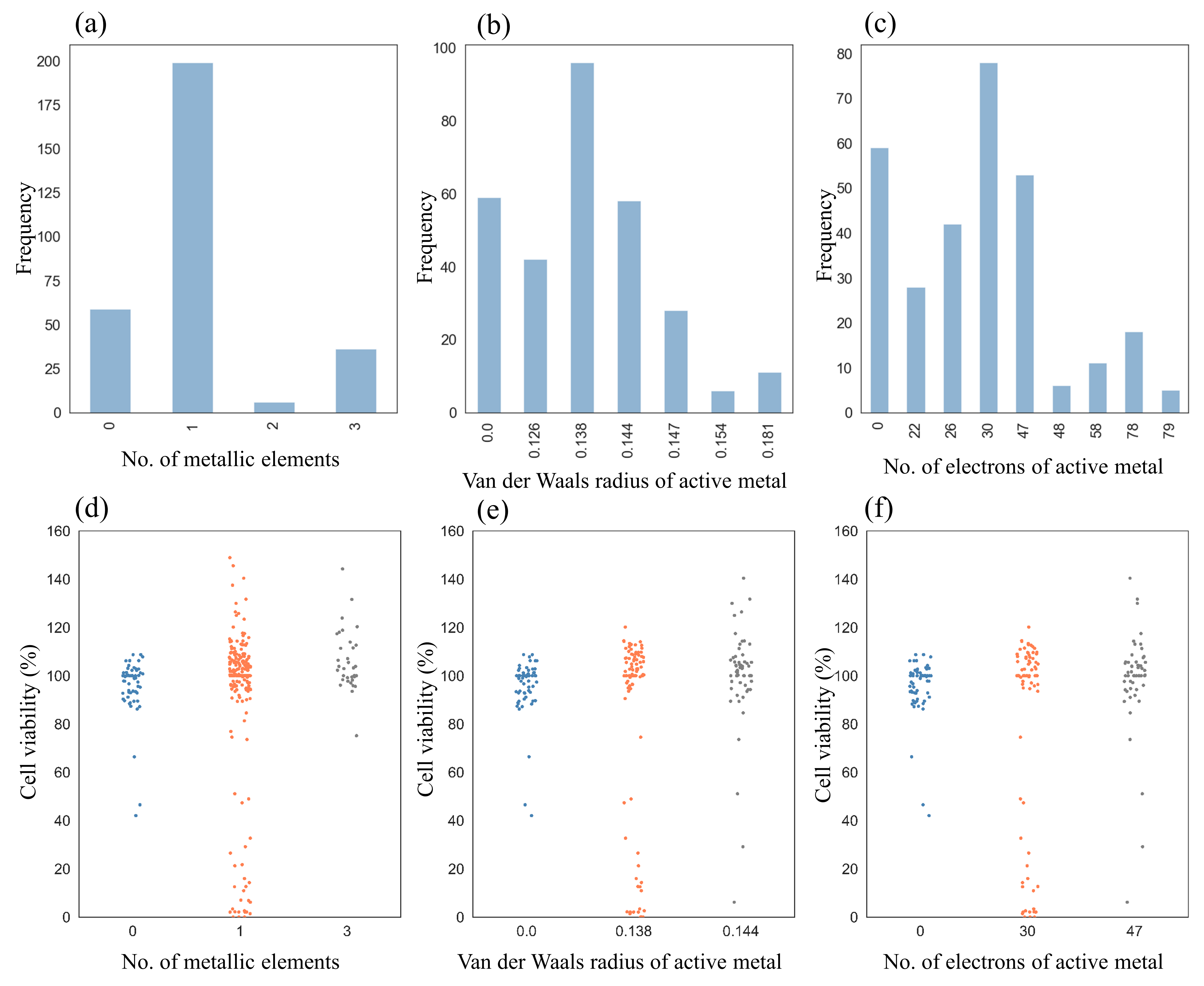


**Figure S4** Dataset overview. (a), (b), and (c) Diversity of a set of nano-descriptors obtained from the Elemental descriptor calculator; (e), (f), and (g) Cell Viability (%) variation trend with representative examples of each nano-descriptor.


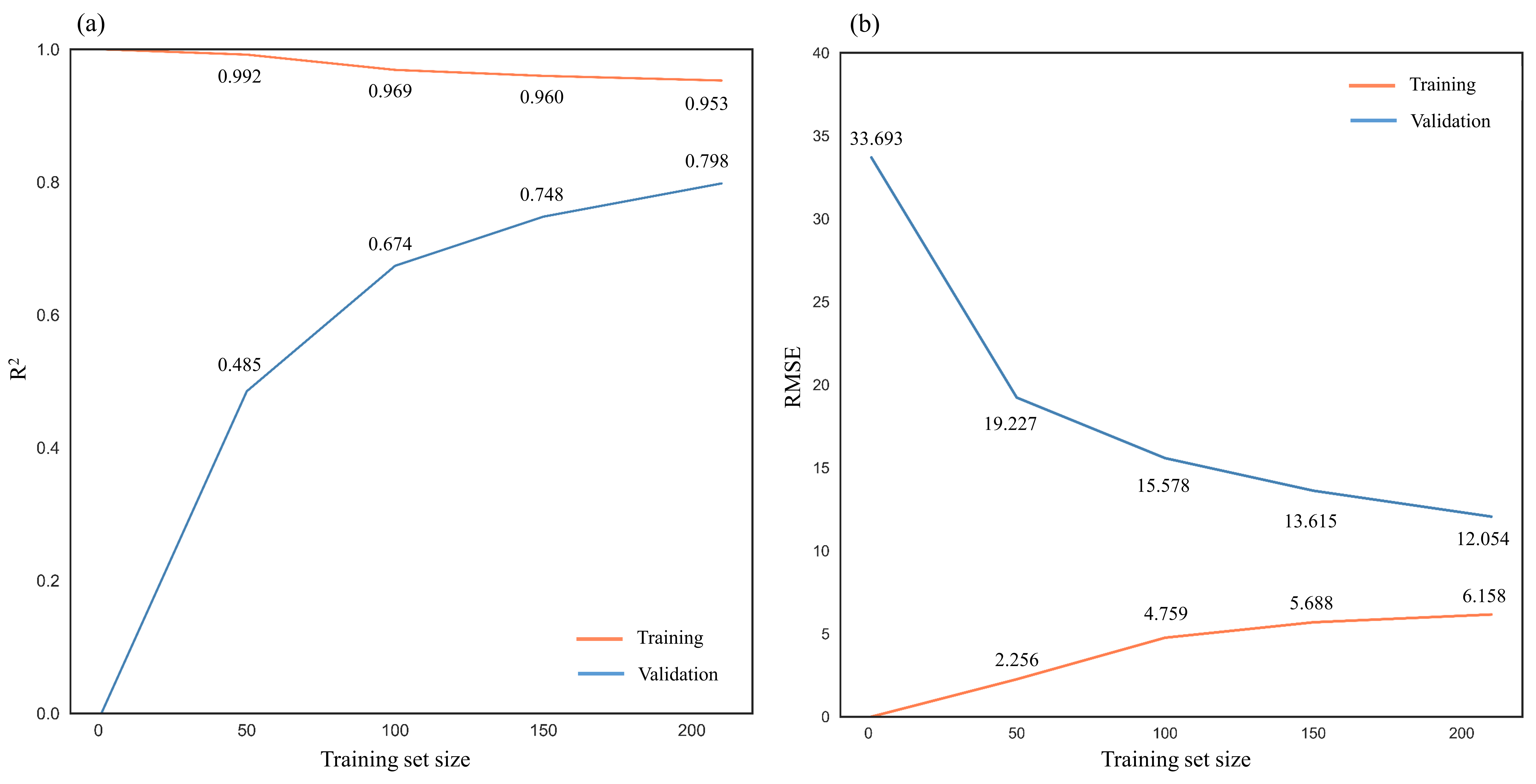


**Figure S5** Learning curves for ET nano-QSTR model for training and validation sets according to (a) determination coefficient (R2) and (b) root-mean-square error (RMSE).

**Table S1** ET nano-QSTR model performance for training, validation, test, and unseen sets assuming data normalization by ENMs surface area.

| **Model** | **Subset** | **R^2^** | **R^2^_ext_** | **Q^2^_LOO_** | **Q^2^_F1_** | **Q^2^_F2_** | **RMSE** | **MAE** | **CCC** |
| --- | --- | --- | --- | --- | --- | --- | --- | --- | --- |
| ET | Training | 0.957 | - | - | - | - | 5.869 | 3.200 | 0.977 |
|  | Validation | 0.725 | - | 0.726 | - | - | 14.329 | 8.361 | 0.844 |
|  | Test | - | 0.761 | - | 0.760 | 0.760 | 13.391 | 7.331 | 0.869 |
|  | Unseen | - | 0.917 | - | 0.921 | 0.918 | 4.625 | 3.316 | 0.957 |
| ET: extra-trees regressor; R^2^: determination coefficient (R^2^_ext_ for external validation); Q: determination coefficient based-metrics (Q^2^_LOO_ for internal validation; Q^2^_F1_ and Q^2^_F2_ for external validation); RMSE: root-mean-square error; MAE: mean absolute error; CCC: coefficient of concordance. | | | | | | | | | |
